# Ballistic study on the penetration potential and injury potential of different bullet types in the use of a newly developed bullet shooting stunner for adequate stunning of heavy cattle

**DOI:** 10.3389/fvets.2023.1143744

**Published:** 2023-03-01

**Authors:** Dominic Gascho, Roger Stephan, Niklaus Zoelch, Michael Vogt, Michelle Aimée Oesch, Michael Thali, Henning Richter

**Affiliations:** ^1^Department of Forensic Medicine and Imaging, Institute of Forensic Medicine, University of Zurich, Zurich, Switzerland; ^2^Institute for Food Safety and Hygiene, Vetsuisse Faculty, University of Zurich, Zurich, Switzerland; ^3^Vogt Waffen AG, Oberglatt, Switzerland; ^4^Scientific Communication and Public Relations, Vetsuisse Faculty, University of Zurich, Zurich, Switzerland; ^5^Diagnostic Imaging Research Unit, Clinic for Diagnostic Imaging, Vetsuisse Faculty, University of Zurich, Zurich, Switzerland

**Keywords:** stunning effectiveness, concussion, animal welfare, slaughter, heavy cattle, *BigBovid*, *Humane Killer*, injury potential

## Abstract

**Introduction:**

Recently, a special bullet shooting stunner for heavy cattle has been developed that fires a bullet instead of a bolt. In the search for a suitable ammunition, the following criteria must be met: First, the energy of the bullet must be sufficient to penetrate the thick frontal bones of heavy cattle. Second, the injury potential at the corresponding penetration depth should preferably be large in order to damage brain tissue relevant to stunning. Third, the bullet must not perforate the occipital bone (over-penetration).

**Methods:**

Four different bullet types [*Hornady FTX, Hydra-Shok, Black Mamba*, and a common full metal jacket (*FMJ*) bullet] were evaluated in a series of experiments on soap blocks and removed bone plates followed by computed tomography examinations. Penetration potential was evaluated in terms of kinetic energy relative to the caliber of the bullet, i.e., mean energy density (*ED*). Injury potential was evaluated by the mean extent of the cavity volume (*e*_*CV*_) at the relevant penetration depth of 5.5 to 7.5 cm in the soap block.

**Results:**

All four bullet types passed through the frontal bone plate. The *ED* was 17.50 J/mm^2^ (*Hornady FTX*), 17.46 J/mm^2^ (*Hydra-Shok*), 13.47 J/mm^2^ (*Black Mamba*), and 13.47 J/mm^2^ (*FMJ*). The *Hornady FTX* and the *Hydra-Shok* each fragmented heavily. The *FMJ* was excluded after three experiments due to over-penetrations. The *e*_*CV*_ was *e*_*CV*_ = 3.77 cm^2^ (*Hornady FTX*), 2.71 cm^2^ (*Hydra-Shok*), and 1.31 cm^2^ (*Black Mamba*), with a significant difference (*p* = 0.006) between the *Hornady FTX* and the *Black Mamba*.

**Discussion:**

For use in heavy cattle, the *Hornady FTX* and the *Hydra-Shok* are recommended due to the larger *e*_*CV*_ than the *Black Mamba*.

## Graphical Abstract

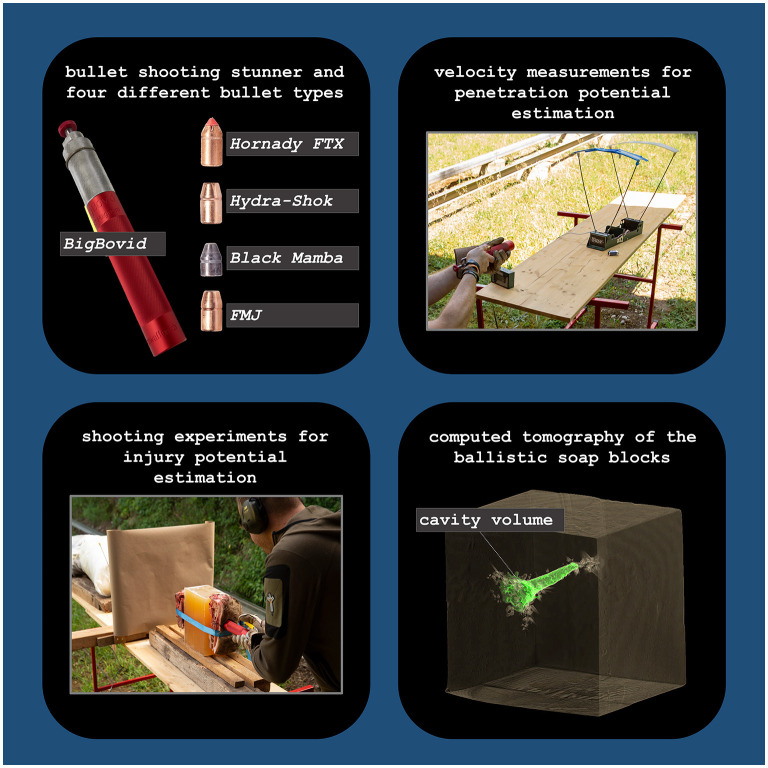

## Introduction

For stunning of cattle, a captive-bolt gun is typically used to damage brain regions that have critical functions for consciousness and the sensation of pain. The goal here is that the animal is no longer sensitive to pain, which is why the term “desensitization” is used for the intended effect of stunning (1). The brain structures to be destroyed are located in the region of the brain stem and thalamus (2), and thus centrally located deep in the brain. A bolt length of 120 mm is sufficient to damage the corresponding brain regions of cattle, except in water buffaloes due to their large frontal sinuses and occasionally in heavy cattle due to their larger skull size (3, 4). In addition, ordinary captive-bolt guns may be insufficient to punch through the frontal bones of heavy cattle with a live weight of up to 1,500 kg, so that adequate stunning is not assured. A previous study (5) has shown that beef bulls and older cull bulls are at higher risk for inferior stunning quality compared to dairy cows, female beef classes, or steer cattle classes. Improper stunning at slaughter causes pain and distress to animals, raising animal welfare concerns (6). To overcome problems in slaughtering water buffaloes and heavy cattle due to the insufficient bolt length and penetrating power of conventional stunning equipment, ordinary handguns are often used (4, 7). Although an improvement in the quality of water buffalo stunning has recently been demonstrated by the use of a pneumatic bolt gun (operating pressure: 200–220 psi; length of ejected bolt: 90 mm) at a newly proposed entry point located 8 cm above the reference point for cattle, a handgun ultimately had to be used in individual cases, even after a second follow-up shot with the bolt (8). In addition, such pneumatic bolt guns are not financially feasible for smaller slaughterhouses.

Since the use of ordinary handguns is not in the sense of our *Federal Food Safety and Veterinary Office* a newly developed 9 mm bullet-shooting stunner, named *BigBovid*, was recently presented for adequate stunning of heavy cattle (3). Instead of a bolt, this device fires 9 mm bullets to damage the corresponding brain regions. In this study (3), two different types of ammunition were used, a light full metal jacket (*FMJ*) truncated cone .3*8 Special* (*Black Mamba*) and a semi-jacketed .3*57 Magnum* with a soft tip (*Hornady FTX*). Based on three velocity measurements and the mass of the bullet, the mean kinetic energy of the respective bullet type when fired with the *BigBovid* was calculated (*Black Mamba*: 527.69 J, *Hornady FTX*: 1133.63 J). As a common measure for the penetration potential in ballistics, the respective energy density (ED), i.e., the kinetic energy per unit reference area, was also given (*Black Mamba*: 8.18 J/mm^2^, *Hornady FTX*: 17.56 J/mm^2^), since this quantity is considered proportional to the penetration depth of a bullet into the tissue (9, 10). For handguns, the caliber squared of the bullet is usually considered the reference surface (9, 10). However, deformation and fragmentation can considerably change the reference area of the bullet, which then affects the penetration depth (9, 10). Since a bullet for adequate stunning of heavy cattle must pass through their thick frontal bones to reach the relevant brain regions, it can be assumed that, with the *Black Mamba* and *Hornady FTX* used, a deformed or fragmented bullet will penetrate the brain tissue. This was shown in the computed tomography (CT) examinations of the severed heads of the heavy cattle, which were stunned with the *BigBovid* (3). The data showed that the *Black Mamba* penetrated deeper into the tissue on average and over-penetrations were observed. The *Hornady FTX* fragmented in each case. Since both types of bullets perforated the frontal bones and reached the relevant brain regions, the recommendation was made for the *Hornady FTX* because of the distribution of fragments and because no over-penetration was observed. However, this alone does not allow a valid statement to be made about the effectiveness of the individual bullet types, since the respective injury potential could not be determined. For a solid recommendation of a bullet type and regulatory endorsement, the potential for injury to the brain regions relevant to stunning is critical. In addition, the bullet type evaluated and finally endorsed should be easily available.

The injury potential of interest depends on how much of the kinetic energy is eventually transferred to the brain tissue at the relevant section of the penetration depth (9, 10). This can be expressed indirectly by the volume of the temporary cavity that is temporarily formed along the bullet path (9, 10). With the extension of this cavity per distance, the deformation forces cause the injury to the tissue, which is stretched and torn in the process. In wound ballistics, this is described as local energy transfer, which is represented by the extent of this cavity (9, 10). Therefore, the extent of the cavity volume at the relevant penetration depth is an indirect measure of the injury potential (9, 10). Glycerin soap is typically used in ballistic experiments as a soft tissue simulant to investigate the extent of the temporary cavity. Such ballistic soap deforms mainly plastically, which means that the maximum extent of the temporary cavity in the soap block is “frozen” and consequently can be well studied and analyzed (10).

The objective of this study was to assess the injury potential of the different bullet types when shot with the *BigBovid* based on the cavities created in ballistic soap after passing through frontal bone plates from heavy cattle. In addition to the bullet types previously used (*Hornady FTX* and *Black Mamba*), two additional bullet types were evaluated in this study. First, the *Hydra-Shok* as an alternative to the *Hornady FTX*, due to its limited availability, and second, an ordinary full metal jacket bullet compared to the full-jacketed *Black Mamba*, in order to point out the increased danger when using an ordinary full metal jacket bullet. For this purpose, shooting experiments were performed and subsequent CT scans of the soap blocks allowed quantitative study of the cavities created by the individual bullet types at the corresponding penetration depth. Accordingly, this study presents the relevant ballistic characteristics of four different bullet types when used with the *BigBovid* and provides an assessment of the injury potential, respectively, the stunning potential when using the *BigBovid* in the slaughter of heavy cattle.

## Materials and methods

### Bullet specifications

**Figure 1:**
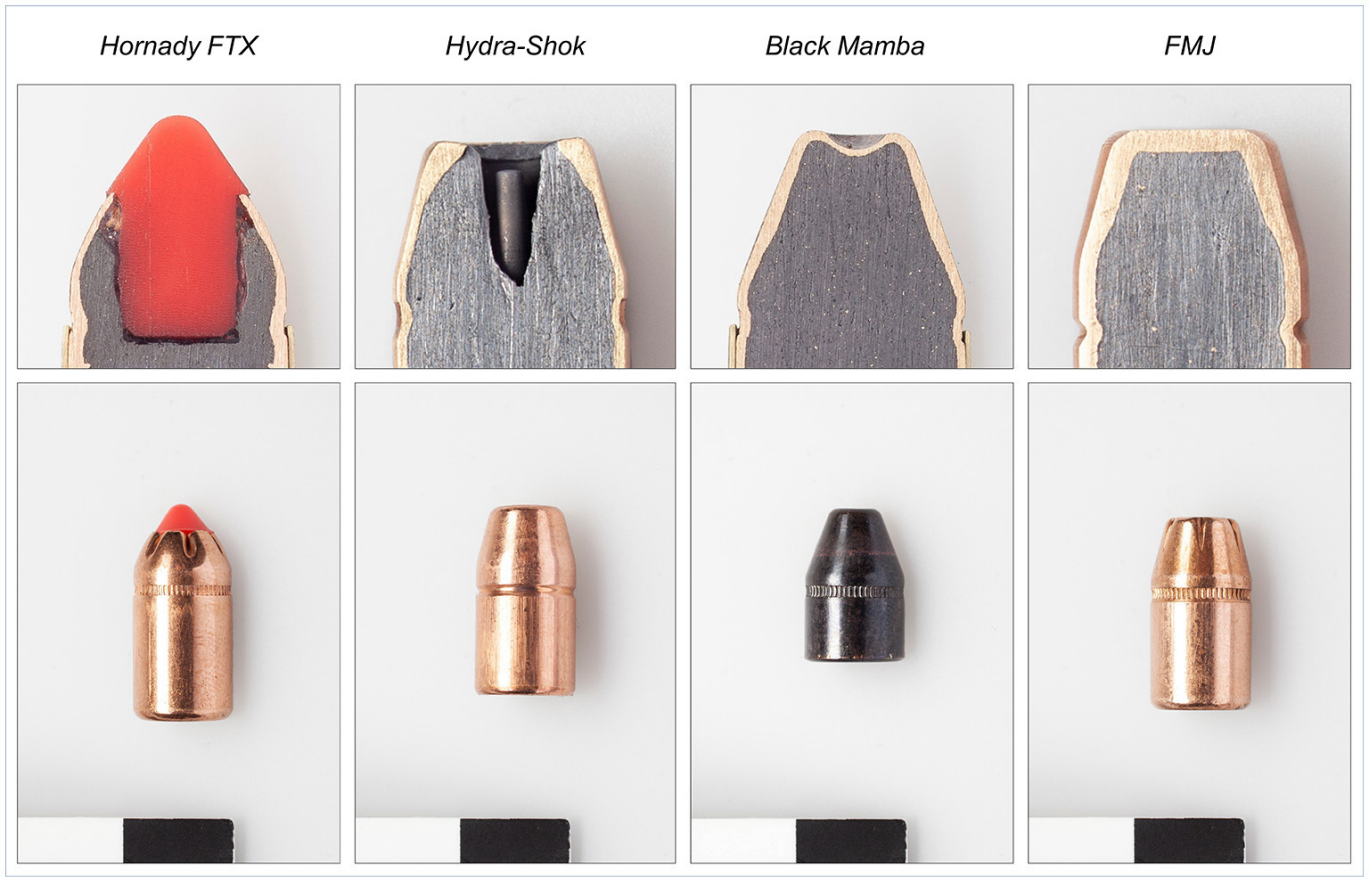
The four different 9 mm bullets evaluated in this study in terms of their injury potential when used with the *BigBovid* for stunning heavy cattle. Note the different design of the tip and the length of each bullet. The *Black Mamba* (110 gr) is shorter and has a lower mass than the *Hornady* (140 gr), the *Hydra-Shok* (158 gr), and the *FMJ* (158 gr).

Four different types of 9 mm bullets were tested for the use with the *BigBovid* bullet-shooting stunner (*Vogt Waffen AG, Oberglatt, Switzerland*). The four bullet types are shown in Figure 1. The first type of bullet was the *Hornady FTX*, a .3*57 Magnum* (*Hornady*^®^ *LEVERevolution*^®^*, Grand Island, Nebraska, U.S.A*.) with a mass of 140 grains (gr) and a special tip (*Flex Tip*^®^ *Technology*) to transfer more energy than conventional bullets with a flat tip. The second type of bullet was the *Hydra-Shok* (*Federal Premium*^®^ *Ammunition, Anoka, Minnesota, U.S.A*.), a readily available ammunition not previously used with the *BigBovid*. This hollow point bullet with a mass of 158 gr and a notched jacket is designed for controlled expansion when penetrating tissue. The third type of bullet was the *Black Mamba*, a full metal jacket truncated cone .3*8 Special* (*Black Mamba, Fiocchi Ammunition, Lecco, Italia*), which has a slight curvature toward the inside at the flat tip and, in addition, this curvature toward the inside also has only a thin layer of the jacket. The *Black Mamba* is an effective hunting bullet with a low mass of 110 gr. The fourth bullet type was a conventional full metal jacket .3*57 Magnum* (*Sellier* and *Bellot, Vlašim, Czech Republic*). This type of bullet, referred to as *FMJ* in this study, was selected to demonstrate potential danger of a conventional full metal jacket bullet when used for stunning cattle in an abattoir. It was assumed that the penetrating depth of this type of bullet with a mass of 158 gr is far too high and over-penetration is very likely. All bullets were fired by one and the same person using the *BigBovid*. The different types of bullets were evaluated in two separate experiment series.

### Experiment series A

The first series of experiments (experiment series A) served to determine the kinetic energy and energy density, the basic data for bullet types used with the gun in question. These quantities were also determined for the bullet types already classified in the earlier study (3), since the data already collected were based on only three velocity measurements.

In the present study, velocities of the bullets were measured at a distance of approximately 50 cm from the barrel using a ballistic chronograph (*Model M-1, Shooting CHRONY Inc., Mississauga, Canada*). This distance was chosen to avoid erroneous measurements due to the expelled propellant gas. In the Supplementary material the experimental setup is illustrated. The measured velocity of the bullet was considered the muzzle velocity, i.e. the velocity the bullet has immediately after leaving the barrel. For each type of bullet ten velocity experiments were performed. From the velocity *v* and the mass *m* of the bullet, the kinetic energy *E* in joule (J) was calculated as follows:

(1A) $$\begin{array}{l} E=\frac{1}{2}\;m{\;v}^{2} \end{array}$$

For the mass of the corresponding bullet type, the value of the manufacturer's weight specification was used in the equation.

In ballistics, the kinetic energy density *ED* (also abbreviated as *KED* or *E*′) in J/mm^2^ is typically expressed as the kinetic energy per cross-sectional area *A*, where the cross-sectional area can be calculated from the caliber of the bullet (*cal*) (10):

(2A) $$\begin{array}{l} d_{\max}\;\sim \;ED=\frac{E}{A}=\frac{2\;m\;v^{2}}{cal^{2}\;\pi \;}. \end{array}$$

For the caliber of all bullet types, a value of 9.07 cm (the conversion value of 0.357 inches rounded to two digits) was used for the calculations of the energy densities.

To account for the propagation of uncertainty in the calculation of the mean kinetic energy $\bar{E}$ and the mean energy density $\bar{ED}$, the mean error of the kinetic energy ($\Delta \bar{E}$), and the mean error of the energy density ($\Delta \bar{ED}$) was determined as follows:

(1B) $$\begin{array}{l} \Delta \bar{E}=\frac{2\;\Delta \bar{v}}{\bar{v}}Ē, \end{array}$$

respectively,

(2B) $$\begin{array}{l} \Delta \bar{ED}=\frac{1}{A}\Delta \bar{E} \end{array}$$

where $\Delta \bar{v}$ is the mean error of the velocity measurements, which was calculated by the standard deviation of the velocities divided by the square root of the number of measurements.

### Experiment series B

In the second series of experiments (experiment series B) the cavity volume created when the bullet penetrated the soap block was investigated. The extent of the cavity over the relevant section of the penetration depth was the measure for the injury potential.

For this purpose, contact shots were performed on ballistic soap blocks (*Mettler-Seifen SA, Henniez, Switzerland*) to which postmortem removed skull bone plates from heavy cattle were attached, namely the frontal bone plate at the front end of the soap block and the corresponding occipital bone plate at its rear end. The soap blocks had a dimension of approximately 25 × 25 × 20 cm and a weight of 13.5 kg. The bone plates were fixed to the soap block with a tension belt. A new pair of bone plates was used for each experiment. For the shot, the *BigBovid* was placed on the frontal bone plate. In the Supplementary material the experimental setup is illustrated. Eight contact shot experiments were intended for each type of bullet. Over-penetration, i.e., penetration of the occipital bone plate and onward flight of the bullet, was considered a major hazard in actual use of the *BigBovid*. Therefore, a bullet type that over-penetrated three times was excluded from further experiments.

After these shot experiments, the soap blocks were packaged and transported to a clinical CT scanner (*SOMATOM*^®^ *Definition Flash, Siemens Healthineers, Erlangen, Germany*), where CT scans of the individual soap blocks were performed. The scan parameters were 120 kV, 400 mAs, and a pitch of 0.35 for reconstructions with an almost isotropic voxel size of approximately 0.6 mm^3^ using a soft kernel (Br38) and a hard kernel (Br60). In addition, reconstructions with extended CT scale were made to visualize the lodged bullets and bullet fragments. The reconstructed CT data were visualized and analyzed using medical image registration software (*syngo.via VB30A_HF07, Siemens Healthineers, Erlangen, Germany*). The total volume (*V*) of this cavity was measured with a region-based image segmentation method (region growing). The penetration depth (*d*) was measured using a linear distance measuring tool.

To estimate the injury potential, the mean extent of the cavity volume along the relevant section of the penetration depth was measured. The relevant penetration depth was derived from mean values of CT-based distance measurements ($\bar{s}$) between the skin and the thalamus ($\bar{s}$ = 10.2 cm) and between the skin to the inner table of the frontal bone ($\bar{s}$ = 3.65 cm) in cattle older than 30 months (4). Taking the difference of the mean distances, the relevant penetration depth from the inner table of the frontal bone to the thalamus was 6.55 cm. Since neither standard deviations of the measurements nor the exact number of CT measurements were given, a deviation of 1 cm was assumed. To estimate the corresponding mean extent of the cavity volume, cross-sectional images with 0.5 cm slice thickness were reconstructed from the CT data along the bullet path starting from the entrance hole. Then, the cross-sectional area (*A*) of the cavity volume was measured on CT images No. 11–15 corresponding to a penetration depth of 5.5–7.5 cm, respectively. A software-based freehand region-of-interest measurement tool was used for these volume measurements. Finally, the mean cross-sectional extent of the cavity volume (*e*_*CV*_) in cm^2^ was calculated along the relevant section of the penetration depth (δ*d*), which is proportional to the injury potential (*IP*):

(3) $$\begin{array}{l} IP\;\sim \;e_{CV}\left(\delta d\right)=\;\frac{1}{n}\;\overset{n}{\underset{i=1}{\sum }}A_{i} \end{array}$$

where *n* is the number of images.

### Statistical analysis

For the measured data (*v*, *V*, *d*) and for *e*_*CV*_(δ*d*) the mean values (standard deviations) are given. For the calculated physical quantities (*E*, *ED*), the mean values (mean errors) are given. The mean values, standard deviations, and mean errors given in this study were rounded to two decimal places.

The Shapiro-Wilk test was used to test for normal distribution. Analysis of variance (ANOVA) was used to test whether the mean values of the bullet types differ from each other in terms *E*, *ED*, *V*, and *e*_*CV*_(δ*d*). When the ANOVA test showed a significant difference, pairwise *t*-tests with *Bonferroni* correction were used as *post-hoc* tests to check between which bullet types there was a significant difference. A *p*-value of <0.05 was considered statistically significant. The *p*-values given are rounded to three decimal places, respectively, very small *p*-values are given as <0.001. An outlier is defined as such if the value is smaller than the first quartile by 1.5 times the interquartile range or larger than the third quartile by 1.5 times the interquartile range. The statistical analyses were done in *RStudio (RStudio, Inc., Boston, MA, USA*).

## Results

Mean values are listed in Table 1 (experiment series A) and Table 2 (experiment series B). Individual values are listed in the Supplementary material. In addition, there are CT reconstructions of all soap blocks in the Supplementary material.

**Table 1:** Results of experiment series A given as mean value (standard deviation) for *v* and mean value (mean error) for *E* and *ED*.

**Type**	***v* (m/s)**	***E* (J)**	***ED* (J/mm^2^)**
*Hornady FTX*	499.13 (7.74)	1130.28 (11.09)	17.50 (0.17)^*^
*Hydra-Shok*	469.32 (6.10)	1127.71 (9.28)	17.46 (0.14)
*Black Mamba*	377.39 (6.78)	507.74 (5.77)	7.86 (0.09)
*FMJ*	412.17 (5.53)	869.79 (7.25)	13.47 (0.11)

*v*, velocity; *E*, kinetic energy; *ED*, energy density.

* w/o outlier: 17.64 (0.11).

**Table 2:** Results of experiment series B given as mean value (standard deviation).

**Type**	***d* (cm)**	***V* (cm^3^)**	***e*_*CV*_(*δd*) (cm^2^)**
*Hornady FTX*	18.50 (2.43)	54.34 (23.39)	3.77 (1.96)^*^
*Hydra-Shok*	17.38 (3.66)	42.13 (17.99)	2.71 (1.41)^**^
*Black Mamba*	18.42 (3.15)	18.31 (5.82)	1.31 (0.33)

*d*, penetration depth; *V*, total volume of the cavity; *e*_*CV*_(δ*d*), cross sectional extent of the cavity volume over the penetration depth section of 9–10.5 cm. w/o outlier:

* 3.22 (1.29),

** 2.30 (0.84).

### Results of experiment series A

**Figure 2:**
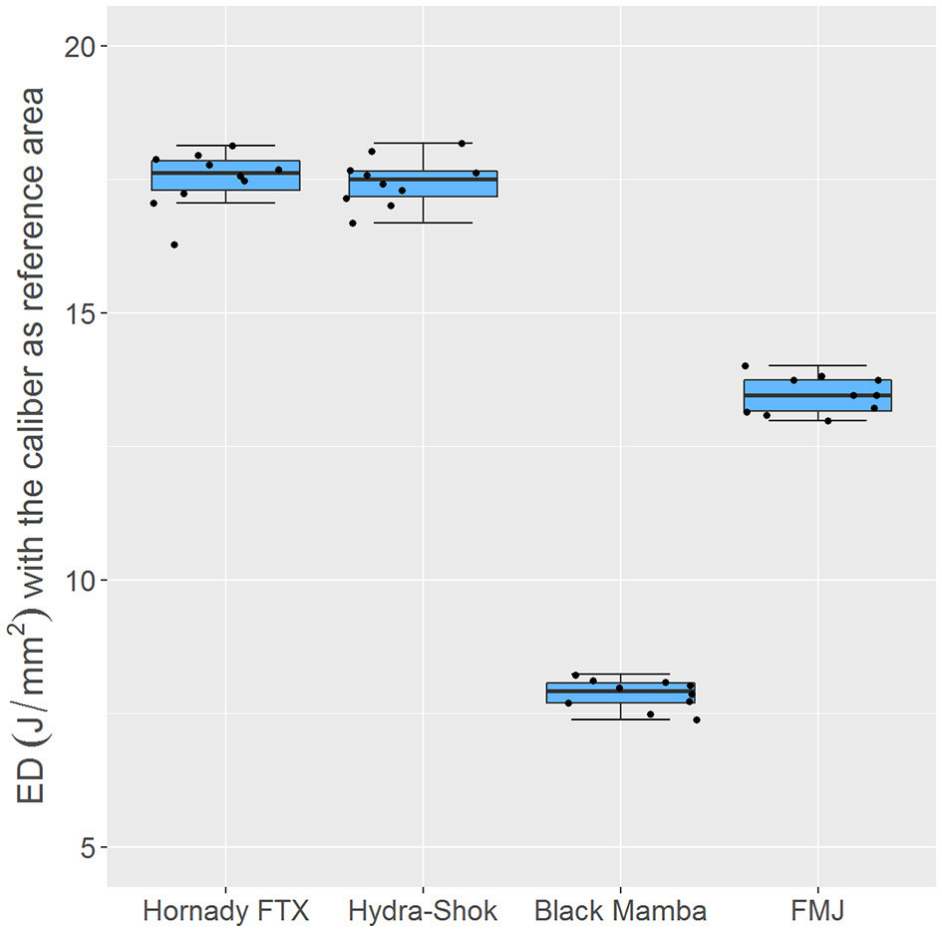
Boxplots of the calculated energy densities in J/mm^2^ for the individual bullet types. The *Hornady FTX* and the *Hydra-Shok* demonstrated almost two and a half times more energy density than the *Black Mamba*. The energy density of the *FMJ* was almost twice as high as that of the *Black Mamba*. There were significant differences (*p* < 0.001) in energy density between the individual bullet types, except between the *Hornady FTX* and the *Hydra-Shok*. The *Hornady FTX* had an outlier with a value of 16.28 J/mm^2^.

With a mean value of 7.86 (0.09) J/mm^2^ the energy density of the *Black Mamba* was more than twice as low as that of the *Hornady FTX* at 17.50 (0.17) J/mm^2^ and that of the *Hydra-Shok* at 17.46 (0.14) J/mm^2^, and almost twice as low as that of the *FMJ* at 13.47 (0.11) J/mm^2^ (Figure 2). The *Hornady FTX* had an outlier with a value of 16.28 J/mm^2^. Excluding this outlier, the Hornady FTX had a mean energy density of 17.64 (0.11) J/mm^2^.

The Shapiro-Wilk test showed that the kinetic energies and the energy densities of the *Hornady FTX*, the *Hydra-Shok*, the *Black Mamba*, and the *FMJ* were normally distributed. The ANOVA test revealed a significant difference between the bullet types in terms of their kinetic energy (*p* = 0.002) and in terms of their energy density (*p* < 0.001). The *post-hoc* tests revealed significant differences (*p* < 0.001) in the kinetic energy and the energy density between the *Black Mamba* and each other bullet type, and between the *FMJ* and each other type of bullet. There were no significant differences between the *Hornady FTX* and the *Hydra-Shok*.

### Results of experiment series B

**Figure 3:**
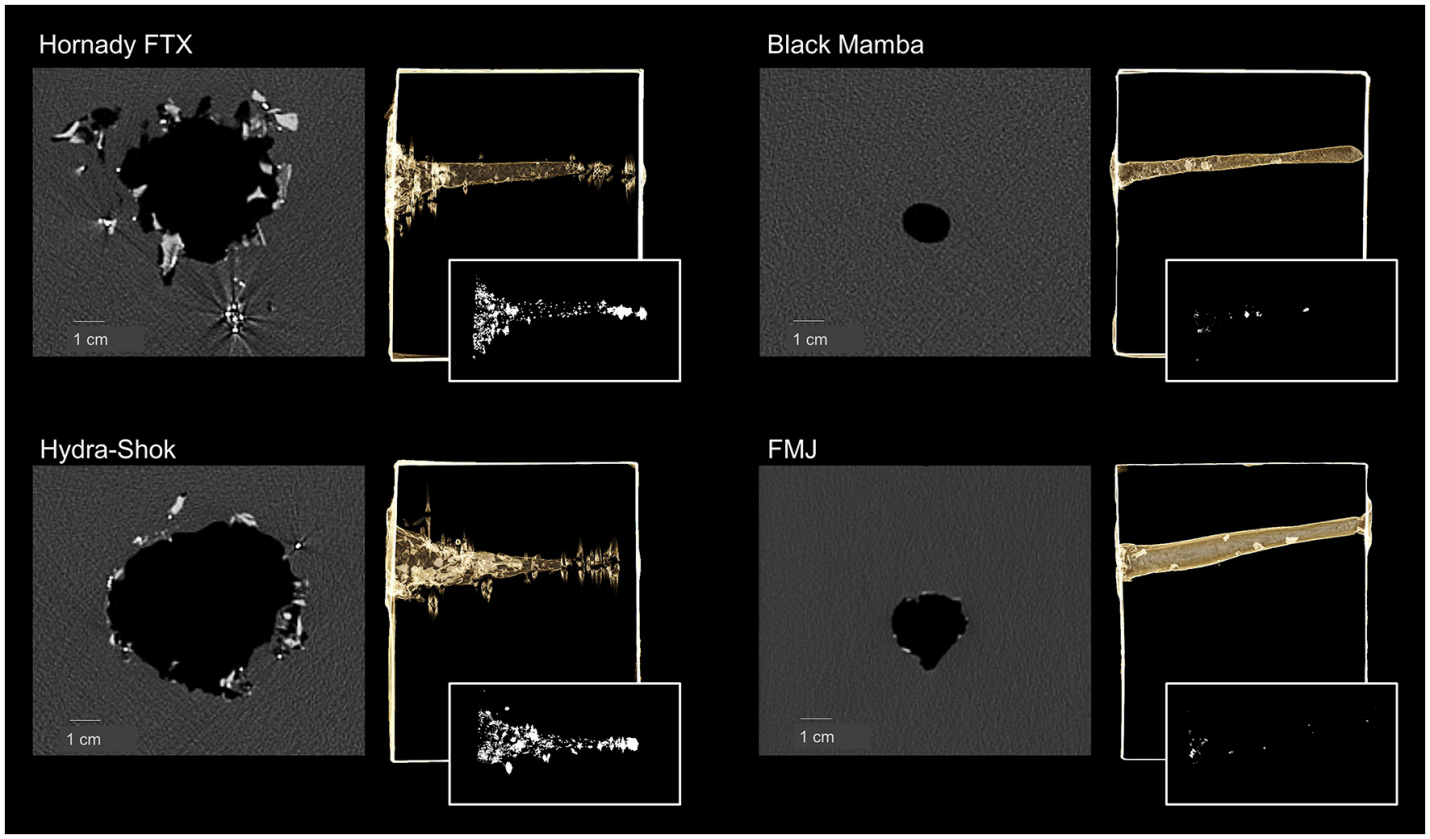
Transverse cross-sectional CT image through the cavity immediately after the perforation of the frontal bone sample and penetration into the soap block (for each bullet type, the left image in grayscale), volume rendering to show the entire cave in the soap block (for each bullet type, the right image kept in the color of the soap block), and maximum intensity projection to highlight bullet fragments along the cavity (for each bullet type, the right small image in black and white).

Soap block shootings were successfully carried out as each bullet perforated the frontal bone plate. However, while for the *Hornady FTX, Hydra-Shok*, and *Black Mamba* all eight experiments were performed without over-penetration, no further experiments were performed for the *FMJ* after three experiments, as this bullet also perforated the occipital bone sample on the back of the soap block. The over-penetrated *FMJ* formed a narrow channel in all three experiments with a total volume of 68.66, 44.89, and 42.76 cm^3^, respectively. After passing through the frontal bone plate only tiny bullet fragments were visible along the narrow channel in the soap block (Figure 3). Due to the small number of experiments with the *FMJ*, this bullet type was excluded from the statistical evaluation.

The *Hornady FTX* and the *Hydra-Sho*k presented numerous fragments along funnel-shaped cavities that started from a large diameter immediately after the frontal bone sample and narrowed as the bullet penetrated the soap block. The *Black Mamba* exhibited few fragments along a narrow channel. The ANOVA test revealed a significant difference between the *Hornady FTX*, the *Hydra-Shok*, and the *Black Mamba* (*p* < 0.001). The mean volume of the *Black Mamba* was significantly smaller than those of the *Hornady FTX* (*p* = 0.001) and the *Hydra-Shok* (*p* = 0.037).

**Figure 4:**
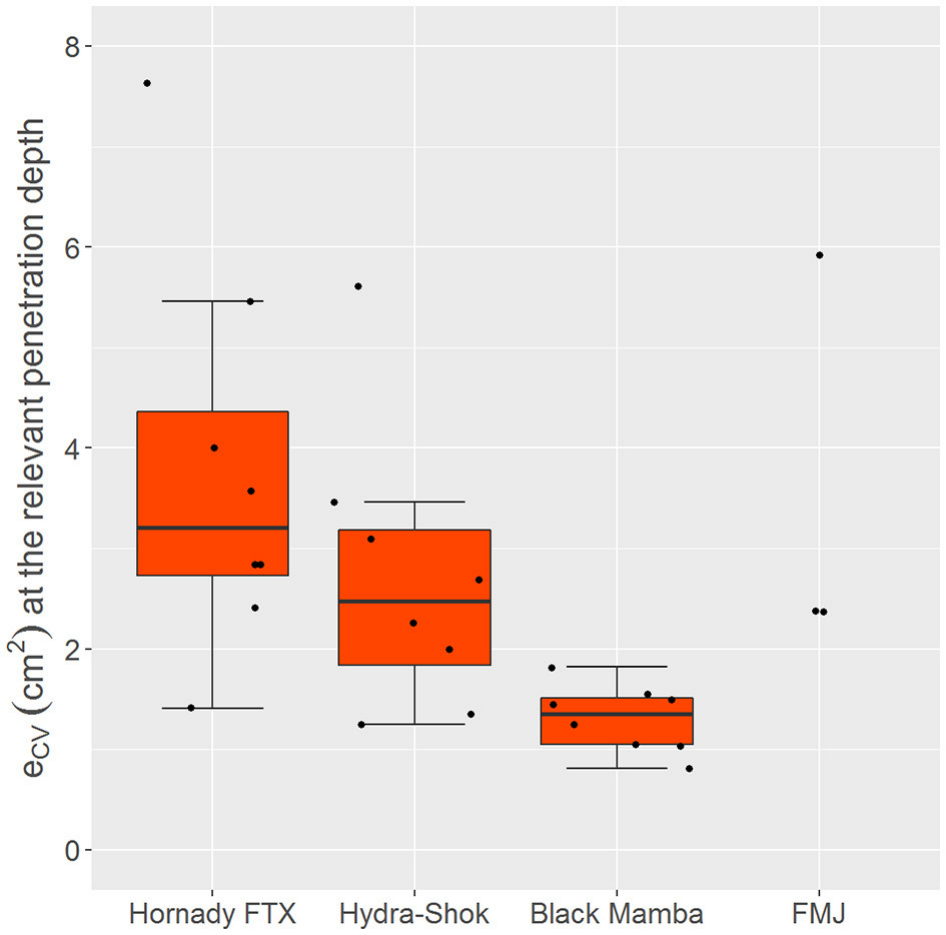
Boxplots of the mean cross-sectional extent of the cavity volume (e_CV_) in cm^2^ along the relevant section of the penetration depth into the soap block (5.5–7.5 cm). The mean volume extent of the *Hornady FTX* and the *Hydra-Shok* was larger than that of the *Black Mamba*. Outlier were observed for the *Hornady FTX* (7.63 cm^2^) and the *Hydra-Shok* (5.61 cm^2^). The *FMJ* achieved values comparable to the *Hydra-Shok*, assuming an outlier at 5.92 cm^2^.

The mean extent of the cavity volume along the relevant section of the penetration depth was 3.77 (1.96) cm^2^ for the *Hornady FTX*, 2.71 cm^2^ (1.41) cm^2^ for the *Hydra-Shok*, and 1.31 (0.33) cm^2^ for the *Black Mamba*. The ANOVA test showed a significant difference between these three bullet types (*p* = 0.002). The *post-hoc* tests revealed a significant difference (*p* = 0.006) in the cross-sectional extent of the cavity volume between the *Hornady FTX* and the *Black Mamba*. The *Hornady FTX* and the *Hydra-Shok* showed outliers with values of 7.63 and 5.61 cm^2^, respectively (Figure 4). After exclusion of the outlier, the mean volume extent was 3.22 (1.29) cm^2^ for the *Hornady FTX* and 2.30 (0.84) cm^2^ for the *Hydra-Shok*. For the *FMJ*, the cross-sectional extents of the cavity volume were 5.92, 2.37, and 2.38 cm^2^.

## Discussion

This study shows the difference between four bullet types when shot with the *BigBovid* through frontal bone plates of heavy cattle into ballistic soap, which allows for assessing the injury potential based on the cross-sectional extent of the cavity volume along the relevant section of penetration depth.

Both, the *Hornady FTX* and the *Hydra-Shok* demonstrated similar mean energy densities. This may seem astonishing at first glance, since the mass of the *Hydra-Shok* was only a factor of 1.13 heavier than the *Hornady FTX*, but the latter reached a velocity almost 30 m/s higher (Δ*v* = 29.81 m/s), and the velocity is squared in the equation for the kinetic energy. In fact, the mean velocity of the *Hydra-Shok* was only a factor of 0.94 less than that of the *Hornady FTX*, and since the velocity is squared in the kinetic energy equation, the differences in mass and velocity equalize in the result, i.e., the mean kinetic energy. Regarding the *Black Mamba*, the low velocity together with the low mass resulted in significantly lower energy values compared to the other three bullet types. The results of the velocity measurements were similar to those of the previous study mentioned in the Section Introduction (3), in which the mean velocity was 499.89 m/s for the *Hornady FTX* and 384.79 m/s for the *Black Mamba*.

The energy density showed the same ratios between the bullet types as the kinetic energy due to the same reference areas, respectively, the same caliber. Consequently, due to the proportionality between energy density and penetration depth, the *Hornady FTX* and the *Hydra-Shok* are expected to penetrate more than twice as far into tissue, assuming that the trajectory of the projectiles is stable. A stable trajectory means that the bullets do not rotate, nor deform or fragment. However, especially due to fragmentation, the penetration depth can decrease considerably. This is mainly due to the strongly decreasing kinetic energy due to fragmentation. A portion of the kinetic energy is converted for the process of fragmentation and remaining kinetic energy is distributed to the individual fragments. From the results of this study, it is obvious that the *Hornady FTX* and the *Hydra-Shok* began to fragment as they penetrated the bone plate, while the other two bullet types were largely unfragmented. Therefore, a large proportion of the kinetic energy has likely been dissipated by the time the *Hornady FTX* and the *Hydra-Shok* penetrated the bone plate. As a result, these two bullet types did not pass through the soap block and eventually perforate the occipital bone plate behind it despite their high energy density, while the *FMJ* with a significantly lower energy density did so in every experiment. The question therefore arises as to the informative value of the energy density for determining the penetration depth through bone structures. In the end, the decisive factor is whether and how much energy was released at the relevant penetration depth, which can be inferred from the extent of the cavity volume.

Based on the extent of the cavity volumes at the relevant penetration depth of 5.5–7.5 cm in the soap block, the injury potential of the *Hornady FTX* and the *Hydra-Shok* is significantly and considerably greater than that of the *Black Mamba*, respectively. The *FMJ* in comparison showed comparable values to the *Hornady FTX* despite the narrow channel. This may seem surprising at first glance, since the extent of the cavity volume of the *Hornady FTX* and *Hydra-Shok* is very large due to fragmentation upon entry into the soap block, but decreases substantially with increasing penetration depth. Despite the comparable injury potential of the *FMJ*, the use of this type of bullet for the stunning of heavy cattle is strongly discouraged because over-penetration was observed in all experiments. In the previous study (3) where the *BigBovid* was used in regular slaughtering, over-penetration was observed even with the *Black Mamba*, although not so in the present study. Consequently, bullets that fragment but reach the relevant penetration depth are preferable for safety reasons.

Safety and proper use are important criteria for the use of a bullet shooting stunner such as the *BigBovid* and therefore a gun purchase license is required. In fact, already at the beginning of the twentieth century stunning devices for cattle have been developed, which discharged a bullet previously inserted into the barrel by striking the firing pin with a wooden hammer; but with these devices, accidents involving gunshot wounds to butchers soon became more frequent, so many butchers were already switching to the safer captive-bolt stunners (11). Today, such bullet shooting stunners are occasionally still in use to stun cattle. A study in the 1980s showed that the *Humane Killer* could fire a 10-g bullet at a velocity of 165 m/s, corresponding to a kinetic energy of 136 J, which was considered sufficient for stunning cattle, based on preliminary studies that showed it would take about 127 J to penetrate the skull of a cattle (12). In this study, a 10-g bullet with 49 lead pellets in a polyethylene casing similar to a shotgun shell was used so that the bullet fragments and the individual lead pellets distribute in the brain, thus preventing over-penetration. Results recently published data (7) showed a kinetic energy of 164 J for an 8-g bullet with a caliber of 7.5 mm when use a *Humane Killer* resulting in an energy density of 3.71 J/mm^2^ (note: in the study an energy density of 2.9 J/mm^2^ is given, which is based on the kinetic energy divided by the caliber squared), which, was enough to pass through the bone plates of water buffaloes, but the bullet had only 8% of its initial kinetic energy left. In the case of heavy cattle, it can therefore be assumed that the *Humane Killer* does not provide the required energy density for the bullet to penetrate the thicker skull plates. Bullet shooting stunners recently developed for water buffaloes (13, 14) are capable of giving a bullet the required energy density, but to our knowledge these are only prototypes so far and are not commercially available. The *BigBovid* used in this study is commercially available and has already shown satisfactory results in the context of regular slaughtering of heavy cattle. In the end, however, it all comes down to the conscientious and practiced handling of the butcher. On the one hand, the butcher must be aware that the stunning device is firing a bullet and, on the other hand, the bullet shooting stunner must be placed at the correct position and operated at the correct angle so that the relevant brain regions are appropriately injured in the process of creating the temporary wound cavity.

In the present study, the cavity volume determined on the CT data was not related to the transferred energy. Although the proportionality factor between cavity volume and energy transferred to the medium is considered to be independent of the shape and size of a metallic projectile when the transferred energy is <2,500 J (10), it is not described how this proportionality between cavity volume and transferred energy behaves when a bullet fragments along its path through the medium. Since both the *Hornady FTX* and *Hydra-Shok* were highly fragmented in the present study, no conversion of the resulting cavity volume to the transferred energy was carried out.

This study has some limitations. First, it should be pointed out that a homogeneous soft tissue simulant was used, which represents tissue very well, but is not identical in its material properties. Although the experimental data of this study are in agreement with the data on the use of the *BigBovid* in the earlier applied study, deviations in routine application cannot be excluded. Second, there are discrepancies between experimental setting and anatomical conditions. Thus, the brain tissue is enclosed by the bony skull and is also smaller in volume compared to the soap block. These discrepancies were considered negligible for the purpose of comparing the four bullet types in terms of their injury potential. Third, the distance between the muzzle of the bullet shooting stunner and the object may show variations in the results. In the event of a contact shot, the propellant gas can penetrate the tissue, contributing to its destruction. At a distance of a few centimeters, this additional effect is lost. In this study, the *BigBovid* was applied directly to the removed forehead plates. However, since the soap block was not fully surrounded by bony structure, the effect of propellant gases may be reduced. Fourth, metal artifacts of the larger fragments on the CT images might influence the segmented cavity volume. However, these deviations in the exact determination of individual volumes are considered negligible in view of the differences in volumes in individual experiments for one and the same bullet type. The method of segmentation itself instead of measuring directly on the soap block is considered accurate. A recent study showed very high accuracy for CT-based determinations of the cavity volume compared to a common method using silicone castings, which turned out to be far less accurate (15).

## Conclusion

Based on the results of this study, we recommend two bullet types for stunning heavy cattle with the *BigBovid*, namely the *Hornady FTX* and the *Hydra-Shok*. First, these bullet types have a high energy density and thus a high penetration potential to pass through the thick frontal bones of heavy cattle. Second, over-penetration is unlikely due to the high fragmentation of the *Hornady FTX* and the *Hydra-Shok*. Third, at the relevant penetration depth, these two bullet types caused extents of the cavity volumes equivalent to and greater than those of the *FMJ* and *Black Mamba*, respectively, and therefore the injury potential of the *Hornady FTX* and *Hydra-Shok* is considered adequate for stunning heavy cattle. Approval of the *BigBovid* with the *Hornady FTX* and *Hydra-Shok* for stunning heavy cattle can now form the basis for further large-scale field studies.

## Data availability statement

The raw data supporting the conclusions of this article will be made available by the authors, without undue reservation.

## Ethics statement

Ethical review and approval was not required for the animal study because no study was performed on live animals. However, we would like to mention here that frontal bone plates of heavy cattle, taken after regular slaughtering, were used for the experiments. No animal was killed for the purpose of the study.

## Author contributions

DG: conceptualization, investigation, methodology, formal analysis, writing and editing, and visualization. RS and NZ: review and editing. MV: investigation. MO: visualization. MT: resources. HR: project administration, conceptualization, investigation, review, and editing. All authors contributed to the article and approved the submitted version.
