# Supplementary material for: Ballistic study on the penetration potential and injury potential of different bullet types in the use of a newly developed bullet shooting stunner for adequate stunning of heavy cattle
Source: Front Vet Sci. 2023 Mar 1;10:1143744. doi: 10.3389/fvets.2023.1143744 (PMC10014789; doi:10.3389/fvets.2023.1143744)
Supplement: Supplementary file 1 [file Data_Sheet_1.docx]

Supplementary material on the experimental setup of the study (original article):

Ballistic study on the penetration

potential and injury potential of

different bullet types in the use of

a newly developed bullet

shooting stunner for adequate

stunning of heavy cattle

*Front. Vet. Sci. 10:1143744.*

doi: 10.3389/fvets.2023.1143744

This is an open-access article distributed under the terms of the Creative Commons Attribution License (CC BY). The use, distribution or reproduction in other forums is permitted, provided the original author(s) and the copyright owner(s) are credited and that the original publication in this journal is cited, in accordance with accepted academic practice. No use, distribution or reproduction is permitted which does not comply with these terms.

Authors:

Dominic GASCHO ^1^*, Roger STEPHAN ^2^, Niklaus ZOELCH ^1^, Michael VOGT ^3^, Michelle Aimée OESCH ^4^, Michael THALI ^1^, Henning RICHTER ^5^

^1^ Department of Forensic Medicine and Imaging, Institute of Forensic Medicine, University of Zurich, Switzerland

^2^ Institute for Food Safety and Hygiene, Vetsuisse Faculty, University of Zurich, Switzerland

^3^ Vogt Waffen AG, Switzerland

^4^ Scientific Communication and Public Relations, Vetsuisse Faculty, University of Zurich, Switzerland

^5^ Diagnostic Imaging Research Unit (DIRU), Clinic for Diagnostic Imaging, Vetsuisse Faculty, University of Zurich, Switzerland

*****dominic.gascho@irm.uzh.ch

Experimental setup: *Experiment series A*

**Velocity measurements**

Velocities of the bullets were measured at a distance of approximately 50 cm from the barrel using a ballistic chronograph. This distance was chosen to avoid erroneous measurements due to the expelled propellant gas.

| 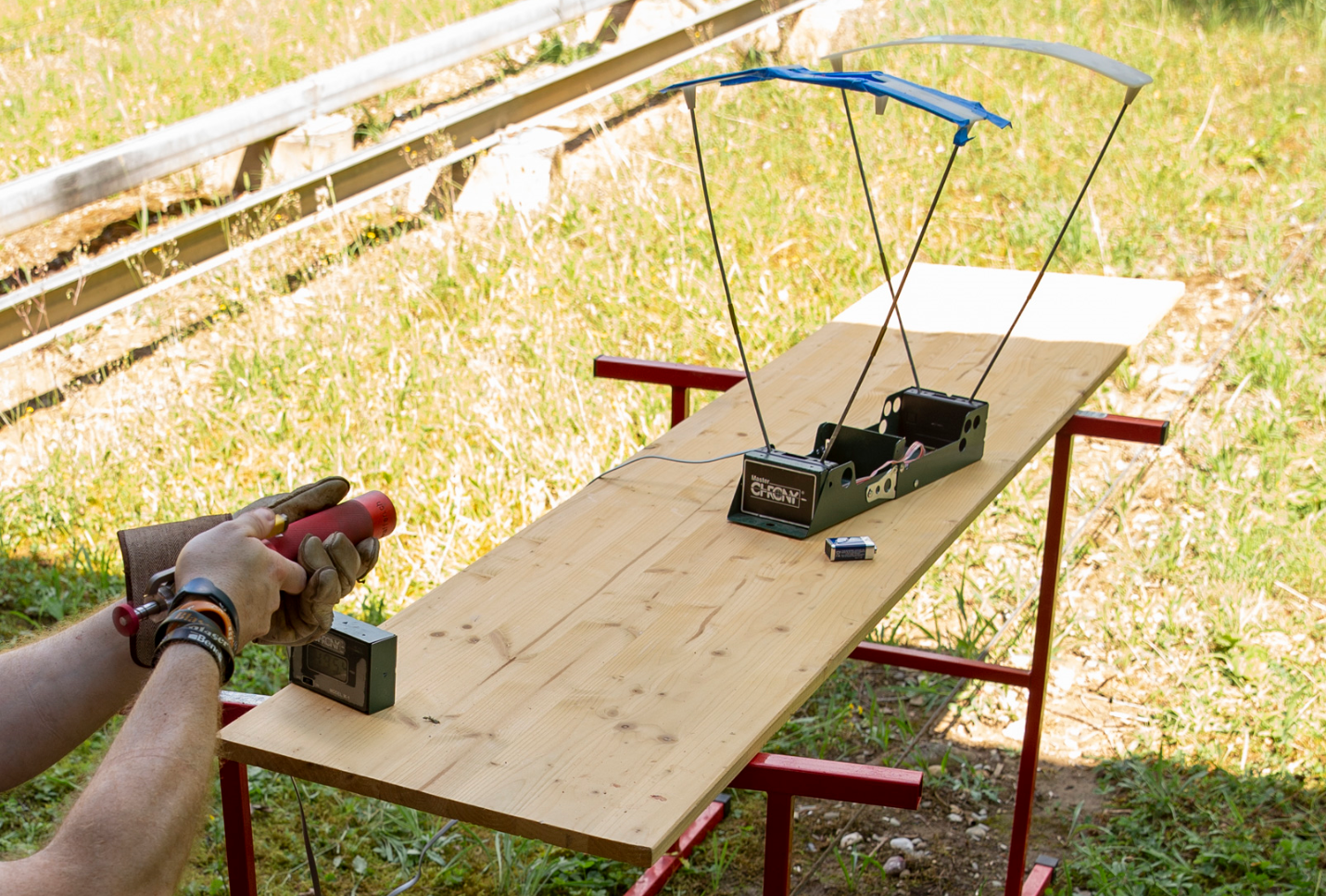 | 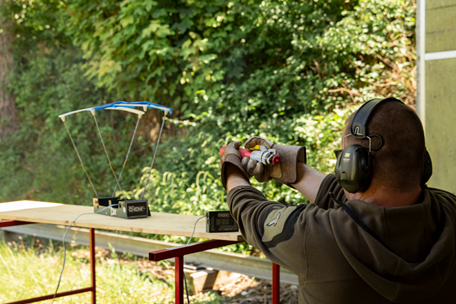 |
| --- | --- |

Experimental setup: *Experiment series B*

**Shots on ballistic soap blocks**

For this purpose, removed frontal bone plates and occipital bone plates of heavy cattle were fixed in front of and behind the soap blocks (25⨯25⨯20 cm^3^) using a tension belt.

| 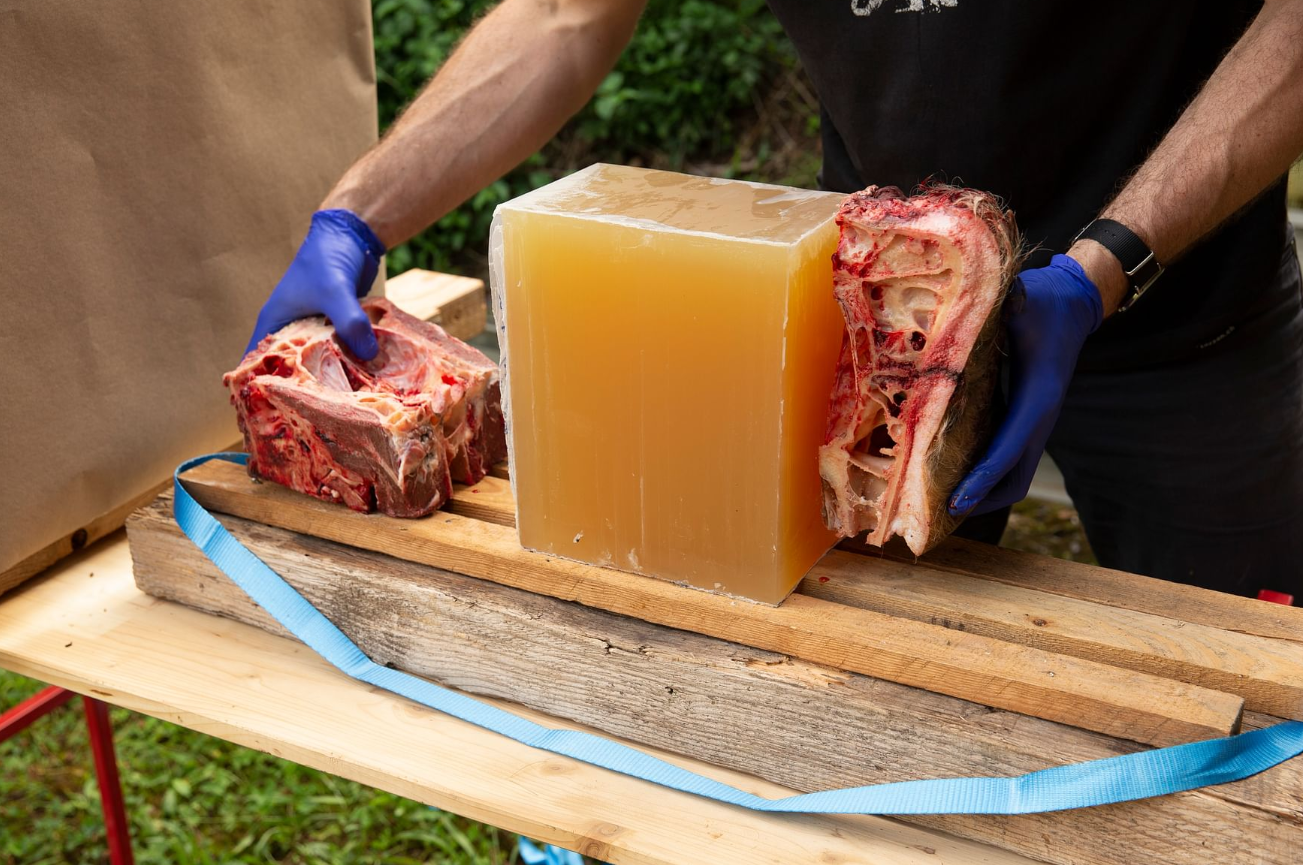 | 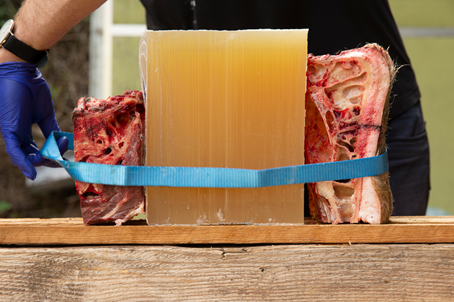 |
| --- | --- |
|  |  |

For the shot experiments, the *BigBovid* was placed about 2 cm off the midline on the frontal bone plate.

| 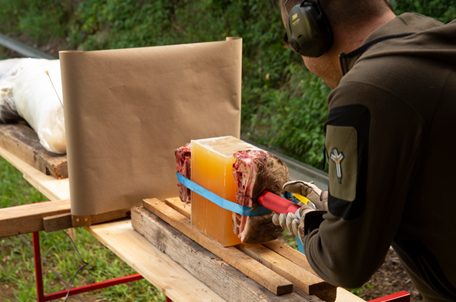 | 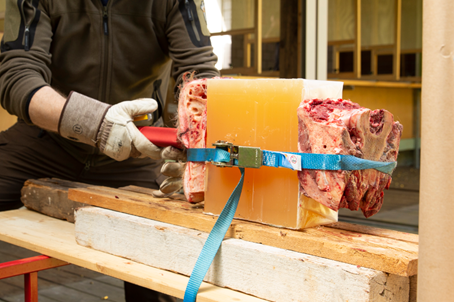 |
| --- | --- |
| 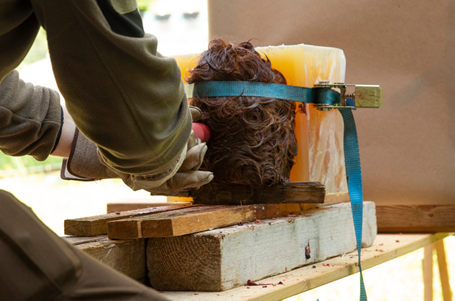 | 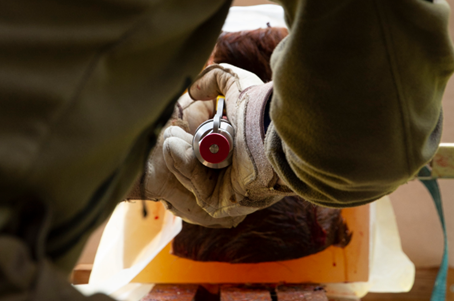 |
