# Supplementary material for: Ballistic study on the penetration potential and injury potential of different bullet types in the use of a newly developed bullet shooting stunner for adequate stunning of heavy cattle
Source: Front Vet Sci. 2023 Mar 1;10:1143744. doi: 10.3389/fvets.2023.1143744 (PMC10014789; doi:10.3389/fvets.2023.1143744)
Supplement: Supplementary file 2 [file Data_Sheet_2.docx]

Supplementary material on the tabulated results of the study (original article):

Ballistic study on the penetration

potential and injury potential of

different bullet types in the use of

a newly developed bullet

shooting stunner for adequate

stunning of heavy cattle

*Front. Vet. Sci. 10:1143744.*

doi: 10.3389/fvets.2023.1143744

This is an open-access article distributed under the terms of the Creative Commons Attribution License (CC BY). The use, distribution or reproduction in other forums is permitted, provided the original author(s) and the copyright owner(s) are credited and that the original publication in this journal is cited, in accordance with accepted academic practice. No use, distribution or reproduction is permitted which does not comply with these terms.

Authors:

Dominic GASCHO ^1^*, Roger STEPHAN ^2^, Niklaus ZOELCH ^1^, Michael VOGT ^3^, Michelle Aimée OESCH ^4^, Michael THALI ^1^, Henning RICHTER ^5^

^1^ Department of Forensic Medicine and Imaging, Institute of Forensic Medicine, University of Zurich, Switzerland

^2^ Institute for Food Safety and Hygiene, Vetsuisse Faculty, University of Zurich, Switzerland

^3^ Vogt Waffen AG, Switzerland

^4^ Scientific Communication and Public Relations, Vetsuisse Faculty, University of Zurich, Switzerland

^5^ Diagnostic Imaging Research Unit (DIRU), Clinic for Diagnostic Imaging, Vetsuisse Faculty, University of Zurich, Switzerland

*****dominic.gascho@irm.uzh.ch

Tabulated Results *Experiment series A*

**Table 1: Results of experiment series A**

| (Series)No. | Type | $v$ [m/s] | $E$ [J] | $ED$ [J/mm^2^] |
| --- | --- | --- | --- | --- |
|  |  |  |  |  |
| (A)01 | *Hornady FTX* | 504.50 | 1154.48 | 17.88 |
| (A)02 | *Hornady FTX* | 505.50 | 1159.07 | 17.95 |
| (A)03 | *Hornady FTX* | 508.00 | 1170.56 | 18.13 |
| (A)04 | *Hornady FTX* | 503.00 | 1147.63 | 17.77 |
| (A)05 | *Hornady FTX* | 495.40 | 1113.21 | 17.24 |
| (A)06 | *Hornady FTX* | 492.80 | 1101.56 | 17.06 |
| (A)07 | *Hornady FTX* | 481.40 | 1051.18 | 16.28 |
| (A)08 | *Hornady FTX* | 501.80 | 1142.16 | 17.69 |
| (A)09 | *Hornady FTX* | 500.20 | 1134.89 | 17.57 |
| (A)10 | *Hornady FTX* | 498.70 | 1128.09 | 17.47 |
|  |  |  |  |  |
| (A)11 | *Hydra-Shok* | 458.70 | 1077.09 | 16.68 |
| (A)12 | *Hydra-Shok* | 470.90 | 1135.14 | 17.58 |
| (A)13 | *Hydra-Shok* | 463.40 | 1099.27 | 17.02 |
| (A)14 | *Hydra-Shok* | 468.80 | 1125.04 | 17.42 |
| (A)15 | *Hydra-Shok* | 478.90 | 1174.04 | 18.18 |
| (A)16 | *Hydra-Shok* | 472.10 | 1140.94 | 17.67 |
| (A)17 | *Hydra-Shok* | 476.80 | 1163.77 | 18.02 |
| (A)18 | *Hydra-Shok* | 467.00 | 1116.42 | 17.29 |
| (A)19 | *Hydra-Shok* | 471.60 | 1138.52 | 17.63 |
| (A)20 | *Hydra-Shok* | 465.00 | 1106.88 | 17.14 |
|  |  |  |  |  |
| (A)21 | *Black Mamba* | 380.40 | 515.72 | 7.99 |
| (A)22 | *Black Mamba* | 365.70 | 476.63 | 7.38 |
| (A)23 | *Black Mamba* | 374.10 | 498.78 | 7.72 |
| (A)24 | *Black Mamba* | 373.50 | 497.18 | 7.70 |
| (A)25 | *Black Mamba* | 381.50 | 518.70 | 8.03 |
| (A)26 | *Black Mamba* | 368.60 | 484.22 | 7.50 |
| (A)27 | *Black Mamba* | 386.30 | 531.84 | 8.24 |
| (A)28 | *Black Mamba* | 382.70 | 521.97 | 8.08 |
| (A)29 | *Black Mamba* | 383.70 | 524.70 | 8.12 |
| (A)30 | *Black Mamba* | 377.40 | 507.61 | 7.86 |
|  |  |  |  |  |
| (A)31 | *FMJ* | 407.20 | 848.81 | 13.14 |
| (A)32 | *FMJ* | 416.40 | 887.60 | 13.74 |
| (A)33 | *FMJ* | 416.40 | 887.60 | 13.74 |
| (A)34 | *FMJ* | 412.10 | 869.36 | 13.46 |
| (A)35 | *FMJ* | 420.60 | 905.59 | 14.02 |
| (A)36 | *FMJ* | 412.00 | 868.94 | 13.46 |
| (A)37 | *FMJ* | 408.40 | 853.82 | 13.22 |
| (A)38 | *FMJ* | 417.60 | 892.72 | 13.82 |
| (A)39 | *FMJ* | 404.70 | 838.42 | 12.98 |
| (A)40 | *FMJ* | 406.30 | 845.06 | 13.09 |
|  |  |  |  |  |

$v$ = velocity, $E$ = kinetic energy, $ED$ = energy density

Tabulated Results *Experiment series B*

**Table 2: Results of experiment series B**

| (Series)No. | Type | $V$ [cm^3^] | $d$ [cm] | $e_{CV}\left( \delta d \right)$ [cm^2^] |
| --- | --- | --- | --- | --- |
|  |  |  |  |  |
| (B)01 | *Hornady FTX* | 13.22 | 16.5 | 1.41 |
| (B)02 | *Hornady FTX* | 55.12 | 19.7 | 2.84 |
| (B)03 | *Hornady FTX* | 56.86 | 20.5 | 4.00 |
| (B)04 | *Hornady FTX* | 61.86 | 13.7 | 5.46 |
| (B)05 | *Hornady FTX* | 31.16 | 20.0 | 2.84 |
| (B)06 | *Hornady FTX* | 88.79 | 19.8 | 7.63 |
| (B)07 | *Hornady FTX* | 54.64 | 20.4 | 3.57 |
| (B)08 | *Hornady FTX* | 73.06 | 17.4 | 2.41 |
|  |  |  |  |  |
| (B)09 | *Hydra-Shok* | 33.70 | 11.8 | 1.35 |
| (B)10 | *Hydra-Shok* | 78.51 | 18.0 | 5.61 |
| (B)11 | *Hydra-Shok* | 27.98 | 19.7 | 3.09 |
| (B)12 | *Hydra-Shok* | 51.88 | 17.8 | 3.46 |
| (B)13 | *Hydra-Shok* | 21.38 | 11.5 | 1.25 |
| (B)14 | *Hydra-Shok* | 48.02 | 20.4 | 2.00 |
| (B)15 | *Hydra-Shok* | 43.79 | 19.9 | 2.69 |
| (B)16 | *Hydra-Shok* | 31.81 | 20.0 | 2.26 |
|  |  |  |  |  |
| (B)17 | *Black Mamba* | 19.65 | 11.6 | 1.03 |
| (B)18 | *Black Mamba* | 21.94 | 20.0 | 1.45 |
| (B)19 | *Black Mamba* | 8.44 | 19.5 | 0.81 |
| (B)20 | *Black Mamba* | 18.58 | 15.7 | 1.50 |
| (B)21 | *Black Mamba* | 19.30 | 20.2 | 1.25 |
| (B)22 | *Black Mamba* | 26.31 | 20.2 | 1.82 |
| (B)23 | *Black Mamba* | 21.16 | 20.1 | 1.55 |
| (B)24 | *Black Mamba* | 11.11 | 20.1 | 1.05 |
|  |  |  |  |  |
| (B)25 | *FMJ* | 68.66 | 20.3 | 5.92 |
| (B)26 | *FMJ* | 44.89 | 20.2 | 2.37 |
| (B)27 | *FMJ* | 42.76 | 20.1 | 2.38 |
|  |  |  |  |  |

$V$ = total volume of the cavity, $d$ = penetration depth, $e_{CV}\left( \delta d \right)$ = the mean cross-sectional extent of the cavity volume along the relevant section of the penetration depth
