# Supplementary material for: Ballistic study on the penetration potential and injury potential of different bullet types in the use of a newly developed bullet shooting stunner for adequate stunning of heavy cattle
Source: Front Vet Sci. 2023 Mar 1;10:1143744. doi: 10.3389/fvets.2023.1143744 (PMC10014789; doi:10.3389/fvets.2023.1143744)

Supplementary material on the CT reconstructions of the study (original article):

Ballistic study on the penetration

potential and injury potential of

different bullet types in the use of

a newly developed bullet

shooting stunner for adequate

stunning of heavy cattle

*Front. Vet. Sci. 10:1143744.*

doi: 10.3389/fvets.2023.1143744

This is an open-access article distributed under the terms of the Creative Commons Attribution License (CC BY). The use, distribution or reproduction in other forums is permitted, provided the original author(s) and the copyright owner(s) are credited and that the original publication in this journal is cited, in accordance with accepted academic practice. No use, distribution or reproduction is permitted which does not comply with these terms.

Authors:

Dominic GASCHO ^1^*, Roger STEPHAN ^2^, Niklaus ZOELCH ^1^, Michael VOGT ^3^, Michelle Aimée OESCH ^4^, Michael THALI ^1^, Henning RICHTER ^5^

^1^ Department of Forensic Medicine and Imaging, Institute of Forensic Medicine, University of Zurich, Switzerland

^2^ Institute for Food Safety and Hygiene, Vetsuisse Faculty, University of Zurich, Switzerland

^3^ Vogt Waffen AG, Switzerland

^4^ Scientific Communication and Public Relations, Vetsuisse Faculty, University of Zurich, Switzerland

^5^ Diagnostic Imaging Research Unit (DIRU), Clinic for Diagnostic Imaging, Vetsuisse Faculty, University of Zurich, Switzerland

*****dominic.gascho@irm.uzh.ch

**Note:**

This supplementary material illustrates computed tomography (CT) data reconstructions of ballistic soap blocks shot with the *BigBovid*, a bullet shooting stunner for heavy cattle, using four different bullet types.

Contact shots were performed on the ballistic soap blocks (Mettler-Seifen SA, Henniez, Switzerland) to which postmortem removed skull bone plates from heavy cattle were attached, namely the frontal bone plate at the front end of the soap block and the corresponding occipital bone plate at its rear end. Bone plates were removed from heavy cattle after regular slaughter. The soap blocks had a dimension of approximately 25 cm by 25 cm by 20 cm and a weight of 13.5 kg. The bone plates were fixed to the soap block with a tension belt. A new pair of bone plates was used for each experiment.

**CT reconstructions: *Hornady FTX*** (Hornady® LEVERevolution®, Grand Island, Nebraska, U.S.A.), a .357 Magnum bullet with a mass of 140 gr and a special tip (Flex Tip® Technology) to transfer more energy than conventional bullets with a flat tip.


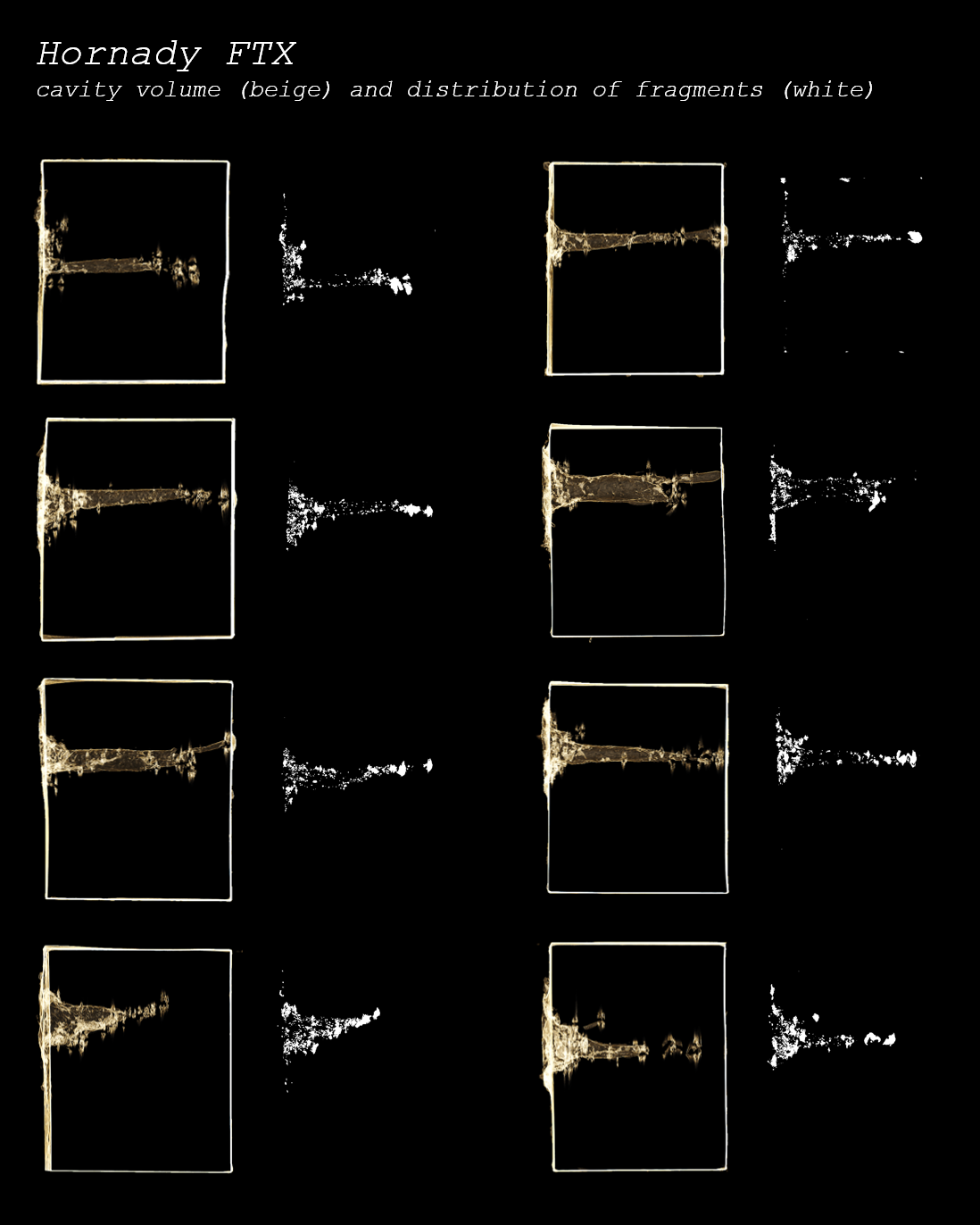


**CT reconstructions: *Hydra-Shok*** (Federal Premium® Ammunition, Anoka, Minnesota, U.S.A.), a .357 Magnum bullet with a mass of 158 gr and a notched jacket that is designed for controlled expansion when penetrating tissue.


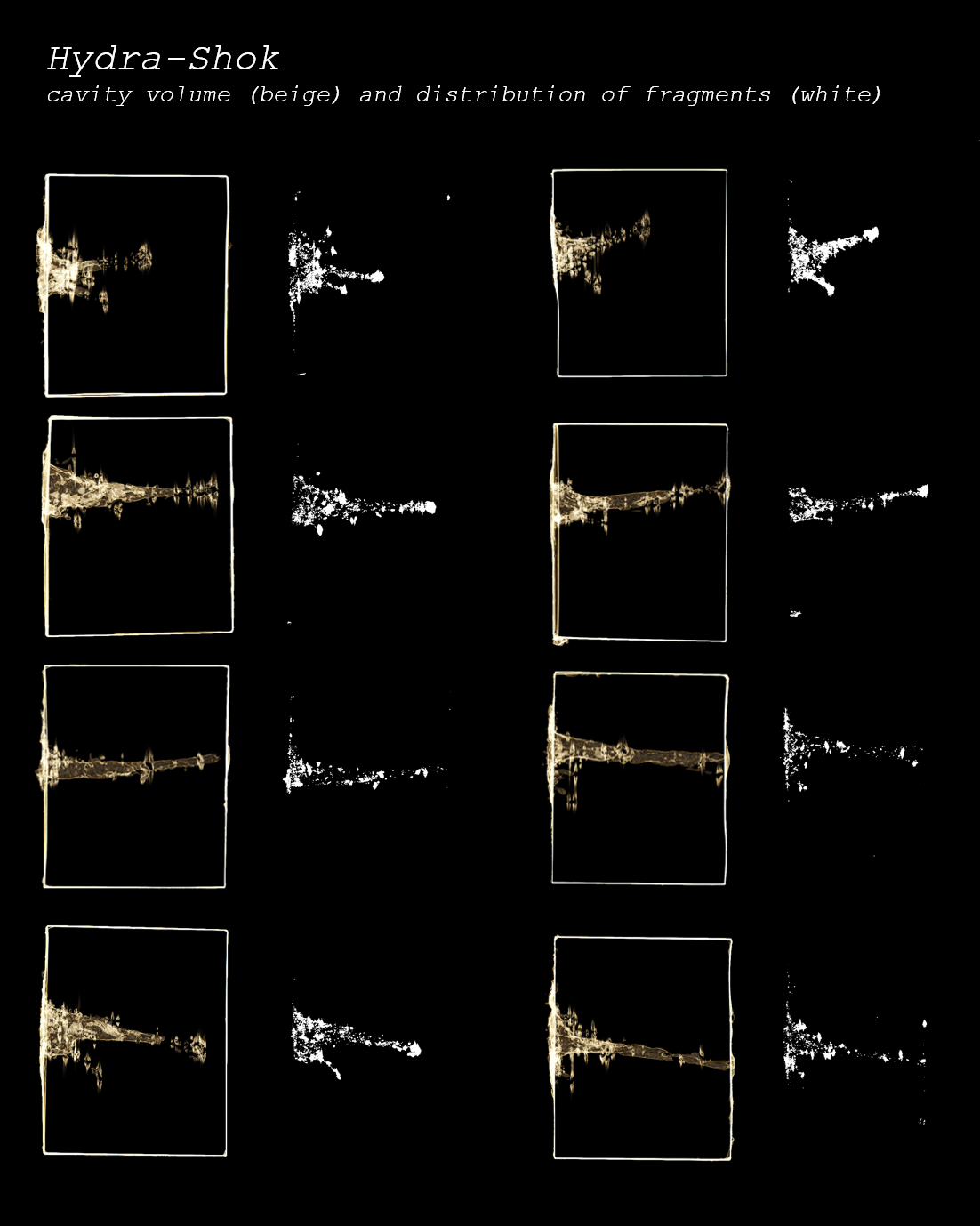


**CT reconstructions: *Black Mamba*** (Fiocchi Ammunition, Lecco, Italia), a full metal jacket .38 Special bullet with a mass of 110 gr, which has a slight curvature towards the inside at the flat tip and, in addition, this curvature towards the inside also has only a thin layer of the jacket.


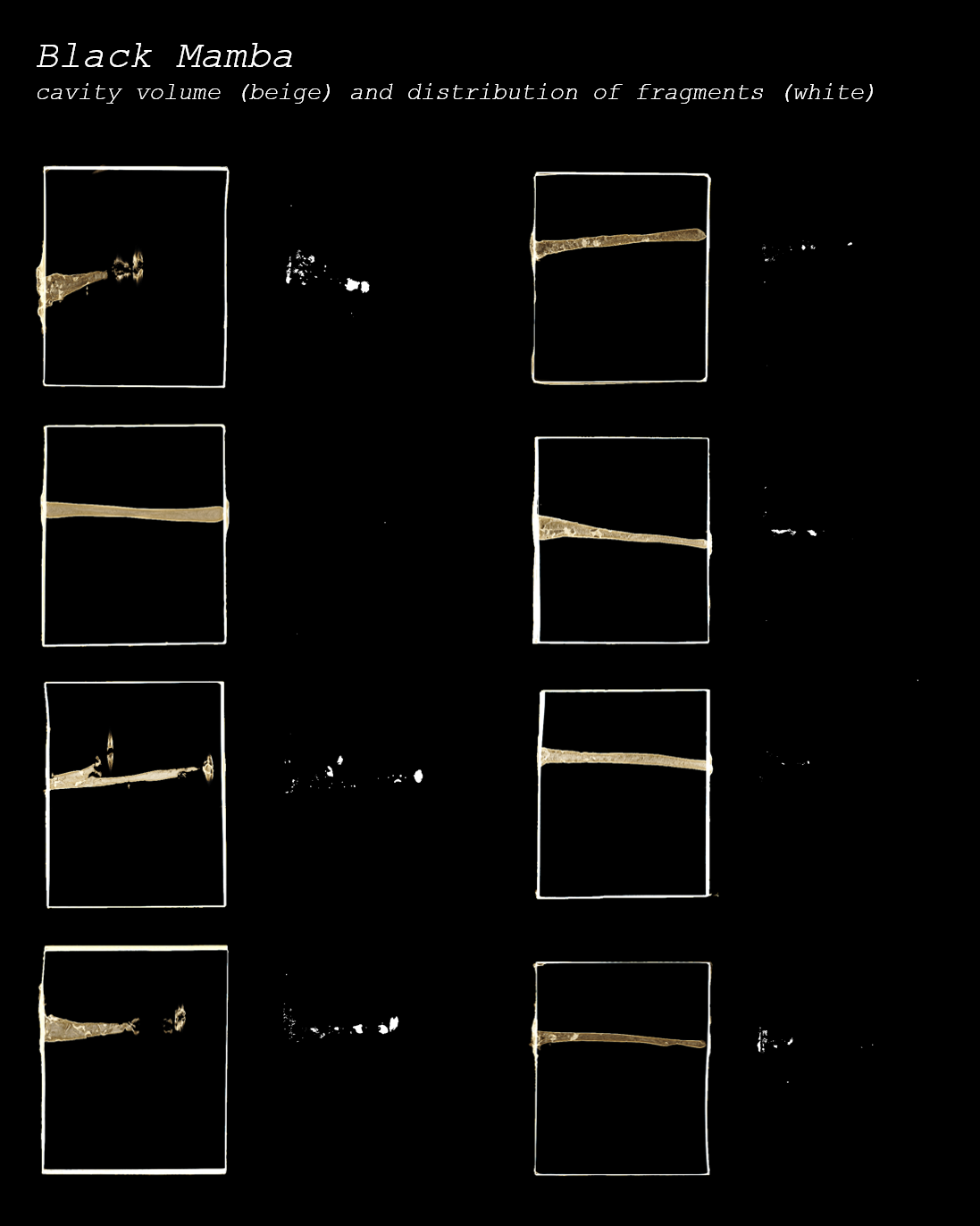


**CT reconstructions: *FMJ*** (Sellier & Bellot, Vlašim, Czech Republic), a common full metal jacket .357 Magnum bullet with a mass of 158 gr. Only three experiments were conducted with this bullet as it also passed through the occipital bone sample on the back of the soap block in each experiment.


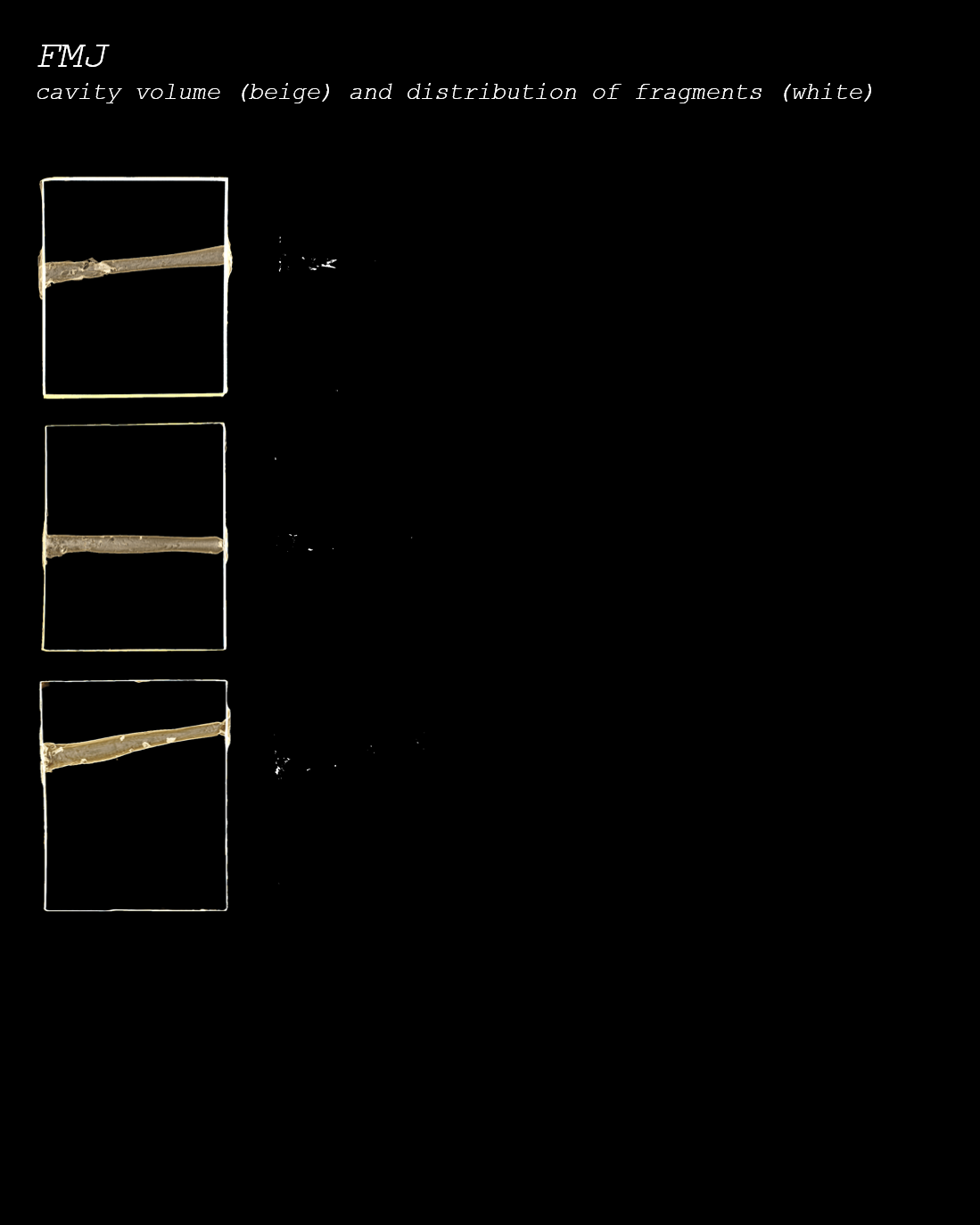

Supplement: Supplementary file 3 [file Data_Sheet_3.docx]
